# Supplementary material for: Direct genome sequencing of respiratory viruses from low viral load clinical specimens using the target capture sequencing technology
Source: Microbiol Spectr. 2024 Oct 14;12(11):e00986-24. doi: 10.1128/spectrum.00986-24 (PMC11537015; doi:10.1128/spectrum.00986-24)
Supplement: Supplemental material — Fig. S1 and S2. [file spectrum.00986-24-s0001.pdf]

Figure S1. Depths of coverage at each position across the SARS-CoV-2 genome normalized to the average coverage obtained in the CS2022-0121 specimen by metagenomic sequencing (A), metagenomic sequencing after depletion of human rRNA (B), target capture sequencing (C), and target capture sequencing after depletion of human rRNA are shown (D). Average coverage is indicated by the dotted line. Areas where viral reads were not obtained are represented by gaps.

Figure S2. Depths of coverage at each position across the 8 segments (PB2, PB1, PA, HA, NP, NA, M, and NS) of the influenza A virus genome normalized to the average coverage obtained in the F16-31-UTM specimen by metagenomic sequencing (A), metagenomic sequencing after depletion of human rRNA (B), target capture sequencing (C), and target capture sequencing after depletion of human rRNA are shown (D). Average coverage is indicated by the dotted line. Areas where viral reads were not obtained are represented by gaps.

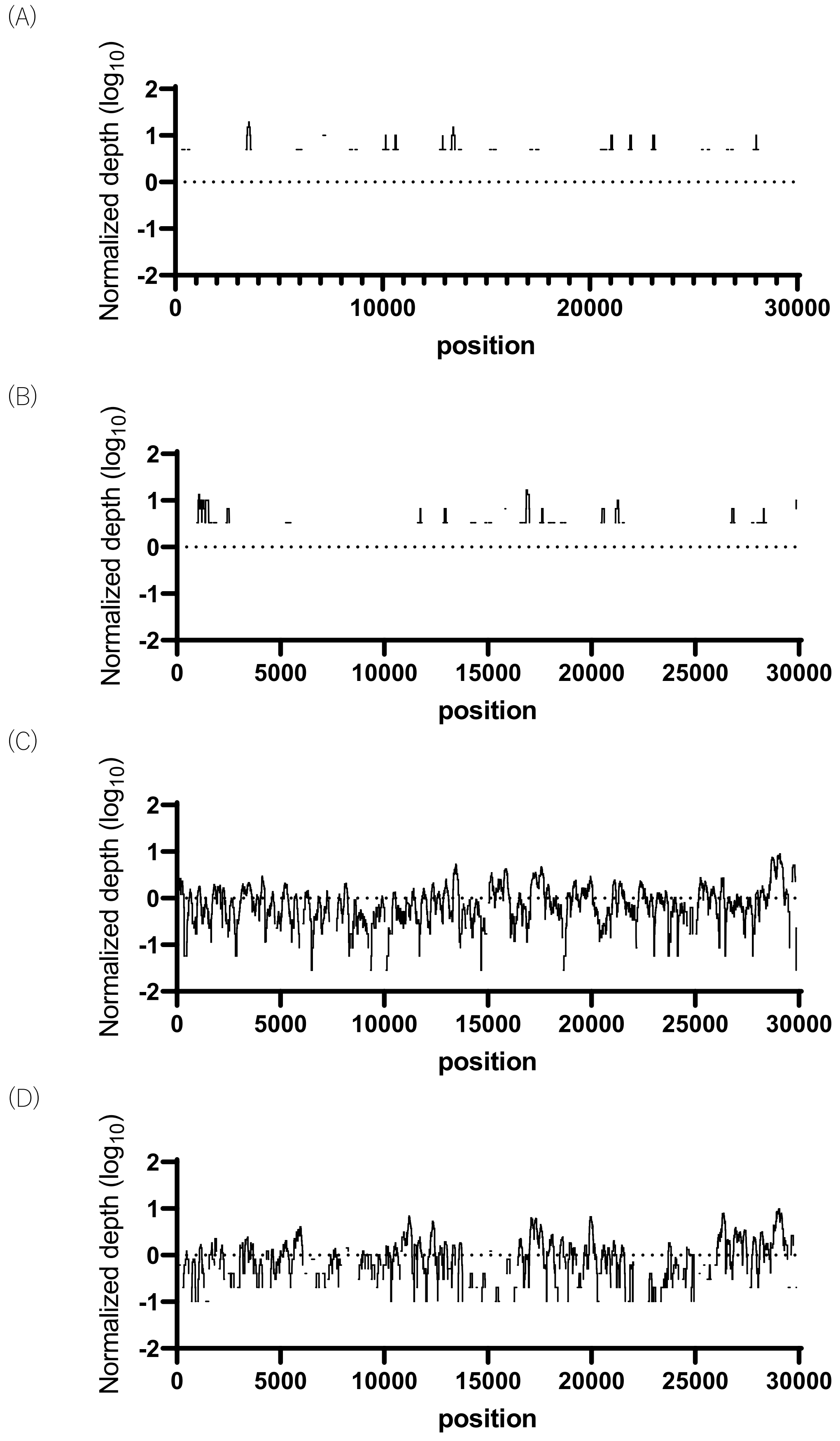
(A)
Normalized depth (log10)
2
1
0
-1
-2
0
10000
20000
30000
position
(B)
Normalized depth (log10)
2
1
0
-1
-2
0
5000
10000
15000
20000
25000
30000
position
(C)
Normalized depth (log10)
2
1
0
-1
-2
0
5000
10000
15000
20000
25000
30000
position
(D)
Normalized depth (log10)
2
1
0
-1
-2
0
5000
10000
15000
20000
25000
30000
position

Figure S1

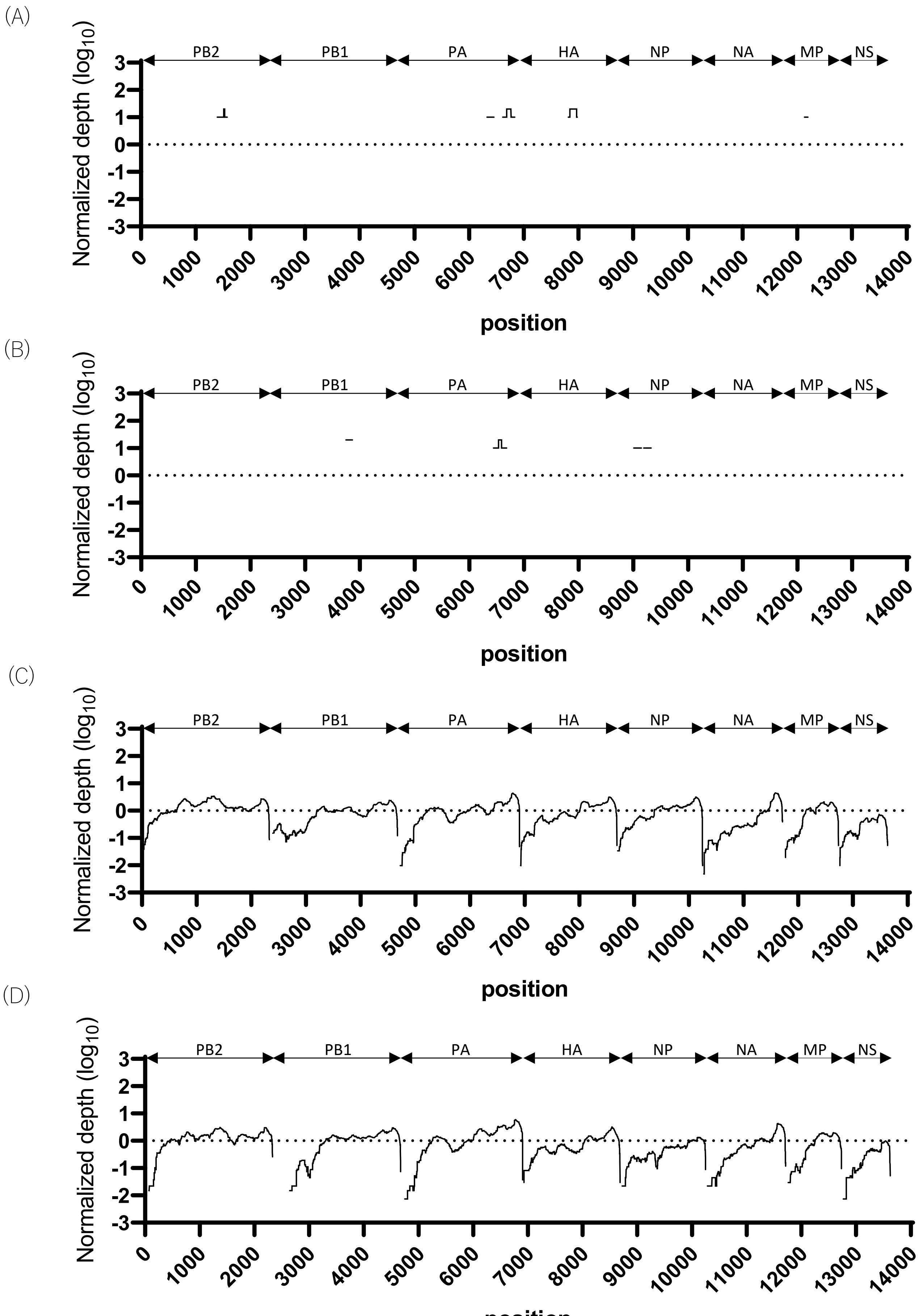
(A)
(B)
(C)
(D)
PB2
PB1
PA
HA
NP
NA
MP
NS
Normalized depth (log10)
3
2
1
0
-1
-2
-3
0
1000
2000
3000
4000
5000
6000
7000
8000
9000
10000
11000
12000
13000
14000
position

Figure S2
